# Protocol of a two arm randomised, multi-centre, 12-month controlled trial: evaluating the impact of a Cognitive Behavioural Therapy (CBT)-based intervention Supporting UPtake and Adherence to antiretrovirals (SUPA) in adults with HIV

**DOI:** 10.1186/s12889-019-6893-z

**Published:** 2019-07-08

**Authors:** R. Horne, E. Glendinning, K. King, T. Chalder, C. Sabin, A. S. Walker, L. J. Campbell, I. Mosweu, J. Anderson, S. Collins, R. Jopling, P. McCrone, H. Leake Date, S. Michie, M. Nelson, N. Perry, J. A. Smith, W. Sseruma, V. Cooper, On behalf of the SUPA Group

**Affiliations:** 1 Department of Practice and Policy, Centre for Behavioural Medicine, UCL School of Pharmacy, Mezzanine Floor, Entrance A, BMA House, Tavistock Square, London, WC1H 9JP UK; 2 Department of Psychological Medicine, Institute of Psychiatry, Psychology and Neuroscience, King’s College London, 16, De Crespigny Park, London, SE5 8AF UK; 3 Centre for Clinical Research, Epidemiology, Modelling and Evaluation, Institute for Global Health, UCL, Royal Free Campus, Rowland Hill Street, London, NW3 2PF UK; 4 MRC Clinical Trials Unit at UCL, 90 High Holborn, 2nd Floor, London, WC1V 6LJ UK; 5 HIV Research Centre, King’s College London, London, SE5 9RJ UK; 6 Institute of Psychiatry at King’s College London, Denmark Hill, London, SE5 8AF UK; 7 Centre for the Study of Sexual Health and HIV, Homerton University Hospital, E9 6RS, London, UK; 8 HIV i-Base, 107 The Maltings, 169 Tower Bridge Road, London, SE1 3LJ UK; 9 Departments of of Pharmacy and HIV Medicine, Brighton & Sussex University Hospitals NHS Trust, Brighton, BN2 5B UK; 10 Department of Clinical, Educational and Health Psychology, UCL, 1-19 Torrington Place, London, WC1E 7HB UK; 11 Kobler Clinic, Chelsea and Westminster Hospital NHS Foundation Trust, 369 Fulham Road, London, SW10 9NH UK; 12 Brighton and Sussex University Hospitals NHS Trust, Brighton, BN2 5BE UK; 13 Department of Psychological Sciences, Birkbeck, University of London, London, UK; 14 UK-CAB, 107 The Maltings, 169 Tower Bridge Road, London, SE1 3LJ UK

**Keywords:** Adherence, Engagement, Antiretroviral therapy, HIV, Randomised controlled trial, Beliefs about medicines, Perceptions, Cognitive behavioural therapy, Motivational interviewing

## Abstract

**Background:**

Delay to start antiretroviral therapy (ART) and nonadherence compromise the health and wellbeing of people living with HIV (PLWH), raise the cost of care and increase risk of transmission to sexual partners. To date, interventions to improve adherence to ART have had limited success, perhaps because they have failed to systematically elicit and address both perceptual and practical barriers to adherence. The primary aim of this study is to determine the efficacy of the Supporting UPtake and Adherence (SUPA) intervention.

**Methods:**

This study comprises 2 phases. Phase 1 is an observational cohort study, in which PLWH who are ART naïve and recommended to take ART by their clinician complete a questionnaire assessing their beliefs about ART over 12 months. Phase 2 is a randomised controlled trial (RCT) nested within the observational cohort study to investigate the effectiveness of the SUPA intervention on adherence to ART. PLWH at risk of nonadherence (based on their beliefs about ART) will be recruited and randomised 1:1 to the intervention (SUPA intervention + usual care) and control (usual care) arms. The SUPA intervention involves 4 tailored treatment support sessions delivered by a Research Nurse utilising a collaborative Cognitive Behavioural Therapy (CBT) and Motivational Interviewing (MI) approach. Sessions are tailored to individual needs and preferences based on the individual patient’s perceptions and practical barriers to ART. An animation series and intervention manual have been developed to communicate a rationale for the personal necessity for ART and illustrate concerns and potential solutions. The primary outcome is adherence to ART measured using Medication Event Monitoring System (MEMS). Three hundred seventy-two patients will be sufficient to detect a 15% difference in adherence with 80% power and an alpha of 0.05. Costs will be compared between intervention and control groups. Costs will be combined with the primary outcome in cost-effectiveness analyses. Quality adjusted life-years (QALYs) will also be estimated over the follow-up period and used in the analyses.

**Discussion:**

The findings will enable patients, healthcare providers and policy makers to make informed decisions about the value of the SUPA intervention.

**Trial registration:**

The trial was retrospectively registered 21/02/2014, ISRCTN35514212.

**Electronic supplementary material:**

The online version of this article (10.1186/s12889-019-6893-z) contains supplementary material, which is available to authorized users.

## Background

Antiretroviral therapy (ART) has resulted in near-normal life expectancy for many people living with HIV (PLWH) [1] and there is solid evidence that viral suppression in PLWH taking ART prevents sexual transmission of HIV [2]. In the UK, uptake of ART among people with diagnosed HIV attending healthcare services is generally high, but a significant number of PLWH are not on ART [3]. The number of people with diagnosed HIV who are not taking ART is likely to be an underestimate because not everyone who is prescribed ART actually receives or takes it [4]. Furthermore, several studies show that nonadherence to ART remains suboptimal: a meta-analysis of 84 studies across 20 different countries found the mean rate of ART adherence (defined as ≥90%) was 62% [5]. In a more recent UK study, 873 (32%) of 2704 people taking ART reported nonadherence [6]. Delay to start ART and nonadherence compromise the health and wellbeing of individual patients, raise the cost of care and impact on public health through increased risk of transmission [7, 8]. Adherence to effective treatment should not only improve physical health, but also psychological wellbeing by empowering patients to take an active role in managing their condition [9]. Interventions to increase adherence to medicines have had limited success and it is not clear which strategies are most effective. Systematic reviews of adherence interventions have reported variable findings [10–13]. A recent recent comprehensive systematic review and meta-analysis of interventions to increase adherence to ART found that a variety of different types of interventions (e.g. interventions delivered via short message service (SMS), counselling and interventions delivered by a treatment supporter) were effective but effect sizes were generally small and the review did not specify specific intervention content or theoretical approaches that were effective [14].

In order to address this, we developed the Supporting Uptake and Adherence to ART (SUPA) intervention. In line with the guidance of the Medical Research Council (MRC) [15], the intervention was informed by our preparatory research in which we explored and developed relevant theory. In our preliminary studies, ART nonadherence had multiple causes for a given individual, both intentional and unintentional [16]. We subsequently identified the salient beliefs about medicines influencing adherence [16]. In studies across a range of illnesses and in different cultural contexts, adherence was consistently related to how patients judged their personal necessity for treatment relative to their concerns about potential adverse effects [17]. Studies conducted with PLWH demonstrated the utility of this Necessity Concerns Framework (NCF) for predicting ART uptake and adherence [18, 19].

In addition to perceptual factors influencing adherence, the National Institute for Clinical Excellence (NICE) recommends addressing practical factors such as limitations in capacity and resources: the Perceptions and Practicalities Approach (PAPA) [20]. To date, few interventions have utilised this approach. One exception is a telephone-based medicines support intervention, where patients receiving the intervention had fewer doubts about necessity and fewer concerns, fewer medication problems and higher reported adherence than standard care controls [21].

The content of the SUPA intervention has been described in detail (King K, Horne R, Cooper V, Glendinning E, Michie S, Chalder T, SUPA Group: The development of an intervention to support uptake and adherence to antiretroviral therapy in people living with HIV: the SUPA intervention, submitted). In summary, the SUPA intervention uses a Perceptions and Practicalities Approach (PAPA) to support uptake and adherence to ART. It comprises 3 key elements aiming to address factors related to the motivation to take treatment, elicit and help the patient overcome barriers to implementing the intention to take treatment and help them establish routines leading to habit formation:

- Communicate a common-sense rationale for ART
- Elicit and address specific Necessity beliefs and Concerns about ART
- Identify and address practical barriers to ART uptake and adherence.

This paper describes the protocol of a study to determine the efficacy of the SUPA intervention for increasing uptake and adherence to ART.

### Primary objective

- To investigate the impact of the SUPA intervention on adherence to ART

### Secondary objectives

- To investigate the impact of the SUPA intervention on treatment outcomes, engagement with care and patient-reported outcomes
- To assess how patients’ beliefs about ART change over time and how this may predict adherence and engagement in care
- To assess the costs and cost effectiveness of providing the intervention in the short and long-term.

## Methods

### Study design

**Fig. 1:**
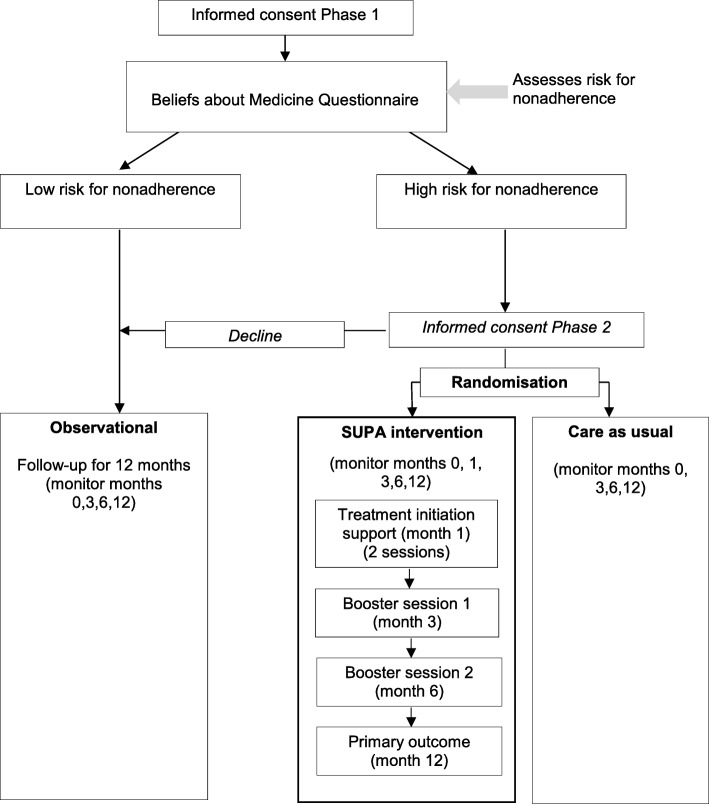
Adapted CONSORT diagram of the study

This study comprises 2 phases (Fig. 1). Phase 1 is an observational cohort study, in which PLWH who are ART naïve and recommended to take ART by their clinician complete a questionnaire assessing their beliefs about ART over a period of 12 months. Phase 2 is a randomised controlled trial (RCT) nested within the observational cohort study to investigate the effectiveness of the SUPA intervention on adherence to ART and to explore the intervention mechanism. PLWH who are at risk of nonadherence will be recruited and randomised 1:1 to the intervention and control arms. All participants will receive usual care which is provided to all NHS patients through clinical support from a multi-disciplinary healthcare team including the patient’s assigned HIV physician, nurses, pharmacists and other healthcare professionals, such as clinical psychologists, as required. In addition, participants in the intervention arm will receive the SUPA intervention.

### Setting

This study will be conducted in HIV clinics in National Health Service (NHS) hospitals across England: Kings College Hospital, Homerton University Hospital, Queen Elizabeth hospital (Woolwich), University Hospital Lewisham, North Middlesex University Hospital, St. George’s Hospital, University Hospitals Birmingham and Bradford Teaching Hospitals. The sites were selected on the basis of clinician-reported issues with suboptimal appointment attendance post HIV diagnosis.

### Participants

#### Inclusion and exclusion criteria

Participant inclusion criteria (Phases 1 and 2): (1) patients aged 18 years or above; (2) diagnosed HIV infection; (3) no previous prescription of ART (except women who have previously been prescribed ART for prevention of transmission during pregnancy and subsequently discontinued, who are considered eligible); (4) clinical recommendation to initiate ART treatment according to contemporaneous British HIV Association (BHIVA) guidelines or as deemed appropriate by the patient’s clinician; (5) able to provide written informed consent and available for long-term follow-up. Participant exclusion criteria: (1) patients who do not speak English; (2) those who plan to leave the country in the next 12 months and hence will not be available for follow-up appointments or telephone follow-ups; (3) participants already enrolled in a trial or in any research study which encourages adherence (e.g. clinical trial of an investigational medical product); (4) lack of capacity to provide informed consent; (5) hospitalisation for a mental disorder in the past 2 years; (6) current suicidality or self-harm; (7) pervasive developmental disorder; (8) active substance misuse/dependence in last 3 months which in the opinion of the physician or investigator renders the patient unable to adhere to the study protocol; (9) patients who have previously started antiretroviral therapy and subsequently discontinued, except if they have previously taken ART for pregnancy; (10) psychiatric or addictive disorders which in the opinion of the clinician or investigator could preclude obtaining informed consent.

### Recruitment

A two-stage enrolment process will be employed: Patients who meet the inclusion criteria will be informed about the study by their HIV doctor, and will subsequently be referred to the study team. All patients who meet the inclusion criteria will be invited to participate in the observational component of the study (Phase 1). Following informed consent, all participants will complete the Beliefs about Medicines Questionnaire-ART specific version (BMQ-ART). The clinician or Research Assistant (RA) will score the questionnaire immediately. If the score indicates that the participant is at high risk for nonadherence (ART-Necessity score ≤ 3 and/or ART-Concerns score ≥ 3), they will be provided with information by the study team and invited to consider taking part in Phase 2, the interventional trial component. Further written informed consent to take part in Phase 2 will be received after the patient has been given an opportunity to consider their participation in the study and discuss this with others if they wish.

### Confidentiality

The study will conform to the Caldicott Principles and ethical and legal guidelines covering consent, confidentiality and storage of data. All data will be kept confidential in accordance with the Data Protection Act. All participants will be allocated a study identification number, so the participants will only be identifiable to study staff. De-identified data (no name, date of birth) will be stored in a password-protected database at the UCL School of Pharmacy for 15 years. After that time the study register, Case Report Forms (CRFs), and consent forms will be destroyed making it impossible to link participants’ names to identification numbers. The original copy of the consent form will be stored in the patient’s case notes and a copy of the consent form will be kept in the site file.

### Randomisation

If the participant consents to participate in Phase 2, they will be randomly allocated to one of the two trial arms (open randomisation; ratio 1:1) by the King’s Clinical Trials Unit (KCTU), King’s College London. The randomisation procedure will be a stratified block randomisation. Centres will be blocked to ensure that there are equal participants in each arm at each site. The randomisation schedule will be incorporated into a dedicated electronic database and kept securely within this electronic system, inaccessible to the enrolment location. Once an eligible participant has given written informed consent and completed the baseline assessment, the RA will log on to the randomisation system, enter the participant identification number and hospital, and will click on the ‘randomise’ function. The treatment allocation will appear immediately on screen and be communicated to the patient. The confirmation of treatment allocation details will be sent to the research team immediately. The RA will inform the participant’s HIV clinical team on the same day. Movement from the observational cohort to the intervention trial or declining participation in the trial will be recorded on the central database.

### Control: care as usual (CAU)

All participants (intervention and control group) will receive care as usual. Although this may vary slightly by clinic, this follows the same framework across all sites.

- If the participant is starting ART, standard of care typically includes:
  - Discussion with a doctor about starting ART (including why, what is involved and the importance of adherence);
  - Consultation with a pharmacist at first prescription discussing the importance of adherence and potential side effects;
  - Collection of 2–4 weeks of medication and appointment with clinic nurse for safety bloods at 2–4 weeks;
  - Review with their HIV doctor at 1 month;
  - Routine clinic visits at 3 or 6 monthly intervals with a nurse and HIV doctor.
- If the participant is not starting ART:
  - 3 monthly consultation with HIV doctor, discussing readiness and beliefs about medication with a view to commencing ART;
  - 3 monthly monitoring bloods with nurse.

### Intervention: SUPA intervention + care as usual (CAU)

The SUPA intervention has been described in detail in a separate publication (King K, Horne, R, Cooper, V, Glendinning E, Michie S, Chalder T: SUPA Group. The development of an intervention to support uptake and adherence to antiretroviral therapy in people living with HIV: the SUPA intervention, submitted). Patients randomised to the intervention group will receive treatment initiation support within 1 month of enrolment into the intervention trial. This support will be in the form of 4 tailored treatment support sessions delivered by a Research Nurse (RN) utilising a collaborative Cognitive Behavioural Therapy (CBT) and Motivational Interviewing (MI) approach. The sessions will be conducted face-to-face (or by telephone if the patient prefers or cannot meet face to face) and will communicate a rationale for the personal necessity of medication, elicit and address concerns about medication and problem-solve potential practical barriers to adherence. Sessions will be tailored to individual needs and preferences, based on (1) the individual patient’s perceptions and practical barriers to ART identified using the BMQ-ART, and (2) salient issues arising during the sessions. An animation series and intervention manual have been developed as tools to communicate a rationale for the personal necessity of ART and to illustrate concerns and potential solutions. The specific timing of the sessions will differ according to the patient’s specific needs and availability; however, the first two sessions will take place within 1 month of enrolment, and sessions 3 and 4 will provide additional support 3 and 6 months post randomisation. These appointments will be scheduled at the patient’s convenience.

### Assessment of intervention fidelity

If the patient consents to audio recording, the intervention sessions will be recorded on a dictaphone. As in previous intervention trials [22], a scale will be developed to rate the recorded sessions according to a rating protocol. Two trained raters working independently will score randomly selected intervention recordings (1 intervention session from 20% of participants). Differences in ratings will be identified, discussed and resolved through a process of consensus and conciliation.

### Participants who delay starting ART (both arms)

If participants delay or decline ART when treatment is offered by their clinician, they will continue in the study. For the intervention group, sessions will focus on barriers to starting treatment, rather than ongoing support with adherence.

### Participant follow-up

#### Phase 1 - observational cohort study

At baseline and at the participant’s routine clinic visits 3, 6, and 12 months post-baseline, the participant will complete the observational study questionnaires (Additional file 1). If the participant does not have 3-monthly appointments as standard care, or are not attending clinical appointments, they will be asked to either schedule a research visit or complete the questionnaires over the phone with a member of the research team.

#### Phase 2 – randomised controlled trial

For trial participants, the baseline visit includes enrolment, randomisation and completion of the trial study questionnaires (Additional file 2). For intervention participants, this includes their first intervention session. Participants in the intervention arm will then be invited to attend additional study visits at 1, 3, 6 and 12-months post-randomisation. Participants in the control arm will be invited to attend additional study visits at 3, 6 and 12 months post-randomisation (Fig. 1). Expenses for study visits will bereimbursed (£10 per visit).

### Data collection

#### Medication event monitoring system (MEMS)

When a participant chooses to initiate treatment, the pharmacy in each site will dispense the participant’s prescription in bottles approved by the SUPA study for use with a Medication Event Monitoring System (MEMS) TrackCap. Site pharmacists will receive training by a designated RA in the use of MEMS caps, and how to explain the use of MEMS cap to participants. The RA will also explain to the participant how to use the MEMS bottle and cap. If the first dispensing of ART in a MEMS bottle does not coincide with a research visit, the RA will contact the participant via telephone to give any necessary further explanation on how to use the MEMS cap. Participants will be offered an instruction sheet which will be given at their research visit. All pharmacy refill data available for each participant from starting treatment up to an including their most recent clinic visit will be collected electronically.

Self-report measures will be completed by the participant with the help of the RA (if desired) at each study visit. All medical data will be collected by the RA from available patient notes and lab results. Baseline data will be collected at the enrolment visit. Measures completed at each assessment are shown in Additional files 1 and 2.

### Primary endpoint

#### The proportion of months under follow-up where adherence is greater than or equal to 90%

Adherence is defined by MEMS data. As the distribution of this variable is unknown, we will categorise the proportion of participants achieving ≥90% adherence as < vs ≥0.8 of the follow-up months. Essentially, this study assesses whether the participant has been ≥90% adherent for at least 80% of their time spent in the trial. The 80% threshold to define a good outcome is based on the fact that 4–6 weeks delay to ART initiation following a treatment recommendation is reasonable, and if followed by consistent ≥90% adherence to ART for the remainder of the trial (10 of the 12 months), the participant is likely to achieve and maintain viral load suppression. To calculate each monthly adherence value, the percentage of adherence (according to MEMS) for each individual over each month of follow-up from randomisation will be calculated, treating every day off ART or not taking all prescribed ART doses (indicating either nonadherence or delayed treatment initiation) as 0% adherence.

### Secondary endpoints

#### Clinical endpoints

##### Treatment failure

Treatment failure is defined as either failure to take up treatment or experiencing virological failure once taking treatment, namely:

- Not starting treatment within 6 months of the treatment recommendation.
- Not obtaining a viral load of < 50 copies/ml 6 months after commencing ART, or following viral suppression to < 50 copies/ml a viral load rebound to > 400 copies/ml on one occasion (single values > 50 copies/ml will be used rather than requiring confirmation because the number of viral load measurements during the 1 year follow-up are too few for confirmation to be possible).
- Following viral suppression to < 50 copies/ml, 2 consecutive viral loads > 50 copies/ml.

##### Disengagement from care at 12 months

Disengagement is defined as missing one or more routinely scheduled visits, including visits either not attended and not rescheduled or rescheduled but not attended before the participant’s next routine appointment is due.

##### Rate of ART regimen switches through 12 months

The total number of drug changes is calculated over the 12-month study period, including changing from one drug to another drug for any reason (excluding changes from 3TC to FTC and vice versa where these are simply due to changing a fixed dose combination tablet).

##### Referral out of the intervention at 12 months

This is defined as being referred out of the intervention for more specialist or intensive care (e.g. seeing a Psychiatrist or Clinical Psychologist for adherence issues). This information will be recorded in clinic notes and monitored by the research team.

#### Patient-reported endpoints

##### Changes in perceptions of ART between baseline and 12 months

Perceptions of ART will be measured using the BMQ-ART [16, 18] which was adapted following preparatory work with the target populations [23] to include items on culturally-specific and practical barriers specific to ART. The BMQ-ART includes 2 scales: the ART-Necessity scale and the ART-Concerns scale. The ART-Necessity scale consists of 10 items which assess how patients perceive their personal need for ART for keeping their HIV under control, maintaining their health and preventing illness. The ART-Concerns scale consists of 10 items measuring concerns about potential adverse effects of ART that have been identified in previous studies. These include fears about short- and long-term side effects, concerns about the timing of tablets and the disruptive effects of the ART regimen on daily life. Participants are asked to rate the extent to which they agree with each item on a scale with possible responses ranging from strongly agree (scored 5) to strongly disagree (scored 1). A total score for each scale is computed by adding the scores for each scale item together and dividing by the number of items. This yields a mean score ranging from 1 to 5 for the necessity and concerns scales.

##### Depression and anxiety at 12 months

The Hospital Anxiety and Depression Scale (HADS) [24] will be used to measure anxiety and depression. This 14-item measure was designed to detect the presence and severity of anxiety and depression among patients attending outpatient clinics without the possibility that scores would be contaminated by reporting of physical symptoms. Possible responses for each item ranged from 0 to 3, with each seven-item scale having a total possible score range of 0 to 21. Higher scores indicate greater anxiety or depression. Good psychometric properties of the HADS have been found in studies including medical outpatients [24, 25].

##### Health-related quality of life at 12 months

The Euroqol-5D (EQ-5D-5 L) is a self-completed measure of health-related quality of life (HRQoL), comprising 5 questions relating to dimensions of health: mobility, self-care, ability to undertake usual activities, pain/discomfort, and anxiety/depression [26]. Each dimension has 5 levels from ‘no problems’ (scored 1) to ‘extreme problems’ (scored 5). This results in a score on each dimension that can be combined to a 5-digit number describing the patient’s state of health. These health states will be combined with population weights to estimate individual utility scores ranging from 0 (worst health) to 1 (full health), required for generating quality-adjusted life years [27, 28]. The scale also includes a visual analogue scale (EQ-VAS) where the participant is required to indicate their level of health on a vertical scale with endpoints labelled ‘the best health you can imagine’ and ‘the worst health you can imagine’. This score (from 0 to 100) represents the patient’s judgement of their own health.

##### Health and social service use at 12 months

Health and social service utilisation will be collected at baseline and each follow-up using a modified version of the Client Service Receipt Inventory (CSRI) [29]. The CSRI is a widely used measure that can be adapted to meet the needs of each study/context in which it is used. It captures retrospective data on accommodation, employment (and time off work), contacts with community health professionals (e.g. GPs, social workers), hospital care (emergency department, inpatient and outpatient), laboratory tests, medication (including ART), social care and informal care from family and friends (i.e. support without payment). The CSRI enquires about whether contacts had occurred, how many, and where appropriate, the duration.

##### Symptoms attributed to having HIV and/or taking ART

Each participant’s experience of symptoms will be measured using the Symptoms Associated with HIV and ART Questionnaire (SAQ) [30]. The SAQ measure consists of 16 symptoms and a section where the participant is invited to add any symptoms that they are experiencing that are not listed. The participant answers by saying whether they experience each symptom or not (yes/no). Where the answer is ‘yes,’ the participant is asked to evaluate the severity of the symptom on a 5-point Likert-type scale with responses ranging from ‘very mild’ to ‘very severe.’ In addition, participants are asked to indicate whether they think the symptom is being caused by HIV, ART, both HIV and ART or neither. Scores will be generated for the total number of symptoms the participant is experiencing (possible range 0–16), the number of symptoms that the participant attributes to HIV (possible range 0–16), and the number of symptoms that the participant attributes to ART side effects (possible range 0–16).

##### Illness perceptions

The 9-item Brief Illness Perception Questionnaire (bIPQ) [31] will be used to assess participants’ cognitive and emotional illness perceptions. Participants are presented with statements about HIV such as ‘how much does your illness affect your life?’. Eight items measure participants’ perceptions of the timeline, consequences, controllability, emotional effects and understanding of their HIV on a scale of 0–10 with anchors relevant to each dimension, where higher scores indicate a greater strength of belief in the particular dimension. The final item asks participants to specify the 3 most important factors that they believe caused their condition. A total score will be calculated by reverse scoring 3 items and adding them to the total score of the remaining 5 items. A higher score reflects a more threatening view of the illness.

##### Self-reported adherence

Adherence to ART is measured using the Medication Adherence Report Scale-5 item version, (MARS-5) [32] consisting of 5 statements about different ways in which the participant might take their medication e.g. ‘I forget to take my medicines,’ scored on a 5-point Likert-type scale, where 1 = always and 5 = never. The statements are introduced in a non-threatening manner in order to minimise social pressure to under-report nonadherence. Adherence is expressed as a continuous scale with possible scores ranging from 5 to 25 with higher scores indicating greater adherence.

##### ART intrusiveness

The ART Intrusiveness Scale [33] consists of 10 questions which assesses the frequency and magnitude that the participants’ medication interferes with different aspects of their lives. This measure consists of 10 statements about ways in which the medication intrudes in their life, e.g. ‘My ART restricts my ability to travel’, scored for frequency on a 5-point Likert-type scale, where 1 = never and 5 = always, and magnitude of the intrusion also on a 5-point Likert-type scale, where 1 = low interference and 5 = high interference. The items are summed for both frequency and magnitude of intrusion and higher scores indicate greater intrusion.

##### Readiness to initiate ART

HIV Treatment Readiness Scale. Following a treatment offer, patients’ perceived readiness to initiate ART will be measured using a single item developed for this study “I feel ready to start antiretroviral medication”. The participant is asked to indicate the extent to which they agree or disagree with this statement on a 5-point Likert-type scale with response anchors “not ready” and “very ready.” This item was created based on the ‘readiness ruler’ [34], a motivational interviewing technique used within the SUPA intervention.

##### Knowledge about HIV treatment

Patients’ knowledge about HIV treatment will be measured using 13 items from the HIV Treatment Knowledge Scale [35]. The original scale consists of 21 items assessing knowledge about HIV and its treatment, e.g. “HIV medications help the body’s immune system get stronger (CD4 increase)”. Eight items were removed either because they assessed knowledge about transmission rather than treatment, or because the topic had been measured in other study questionnaires. Participants are required to indicate whether they believe each statement is true or false or whether they do not know the answer. A total score is calculated by summing the number of correct responses and dividing by the total number of scale items. This yields a percentage of correct responses, with higher scores indicating greater HIV treatment knowledge.

### Qualitative data

A subset of at least 20 participants receiving the SUPA intervention will be interviewed by a RA within 3 months of receiving the last intervention session. To avoid bias, the RA will be independent to the RN delivering the intervention. The interview schedule will explore participants’ perceptions of the intervention (e.g. overall impression, positive features, room for improvement, ease of comprehension, perceived effect on adherence and overall wellbeing). Transcripts will be subjected to thematic analysis.

### Sample size calculations

#### Phase 1 - observational cohort

Our risk assessment for nonadherence was based on the findings of a previous prospective study of beliefs and adherence among PLWH [19]. Phase 1 assesses whether the initial risk assessment is valid in the current sample. As such we will not aim to obtain a particular sample size, but rather recruit as many patients as possible (all eligible patients attending the clinic will be invited to Phase 1).

#### Phase 2 – trial

Since this study uses a novel measure combining both uptake and adherence, there are no data to inform a sample size calculation. It is plausible that the distribution could be bimodal or highly skewed. As the proportion with ≥90% adherence will also be bounded by [0,1], standard sample size calculations based on the normal distribution would likely be inappropriate, even if a standard deviation could be hypothesised. This study therefore defines a good primary outcome as ≥80% of follow-up months with ≥90% adherence. Since the study selects for an at-risk group, we would expect a large difference between control and intervention groups, and a 15% difference between groups is considered clinically significant based on estimated intervention costs. Table 1 shows the number of participants needed in each arm to detect a 15% difference in adherence from a range of possible control group percentages with > = 0.8 of follow-up months with ≥90% adherence (80% power, 2-sided alpha = 0.05). We will therefore recruit 372 participants.

**Table 1:** Sample size calculations for Phase 2 – trial

Standard of care group % with > 0.8 of follow-up months with ≥90% adherence	Intervention group % with > 0.8 of follow-up months with ≥90% adherence	N per arm	Total N
35%	50%	183	366
45%	60%	186	372
50%	65%	183	366
60%	75%	165	330
70%	85%	134	268

### Statistical methods and analysis

The SUPA intervention is hypothesised to be superior to care as usual, and therefore the planned analysis is intention to treat, including all randomised participants, with all participants analysed according to the study group to which they were randomised regardless of subsequent treatment received. Primary analysis will include all randomised participants other than those randomised in error (defined as not intending to randomise the participant through e.g. miscommunication, rather than a participant or clinician decision once the allocation has been given).

A per-protocol analysis will be carried out on the primary endpoint including all participants in the intervention group who attended all 4 (2 + 1 + 1) sessions. If the intention to treat and per-protocol analyses on the primary endpoint leads to inconsistent results, then per-protocol analysis will also be carried out on all the other endpoints.

Continuous variables will be summarised by medians and IQRs or means and standard deviations as appropriate depending on the distribution, and compared between groups using ranksum tests or t-tests respectively. Comparisons of change from baseline in continuous variables will adjust for any baseline imbalances using either quantile or normal linear regression (depending on the shape of the distribution).

Categorical variables will be summarised by frequency tables, and compared between groups using chi-squared tests, unless any cell count is < 5 or cell percentage is < 5% in which case exact tests will be used. Binary variables will be summarised by percentages, using standard exact 95% CI for the risk differences. Time-to-event variables will be summarised using Kaplan-Meier curves and average differences between randomised groups estimated using Cox models. Patients without the event recorded will be censored at their last clinic visit. Proportionality of hazards will be tested; where significant departures exist, varying differences between randomised groups over time will be estimated using flexible parametric models of Royston and Parmar. Rate of treatment switching will be analysed using Poisson regression, including all changes to ART as events and the total time under follow-up through the earliest of 12 months or the last patient visit as the person-time at risk. Primary analysis will not stratify by clinical centre.

### Subgroup analyses

Subgroup analyses will be performed to assess heterogeneity in differences between randomised groups for the primary endpoint according to gender, ethnicity, number of intervention sessions attended, early (treatment indicated at point of diagnosis) vs late diagnosis (treatment not indicated at point of diagnosis), starting for clinical need vs starting for treatment as prevention, baseline CD4 count, and baseline BMQ scores - low Necessity vs high Concerns vs both low Necessity and high Concerns. Subgroup analyses will use logistic regression to model interactions between randomised group and the factors above.

### Health economic analyses

#### Perspective

The evaluation will primarily adopt a health and social care perspective as preferred by the National Institute for Health and Care Excellence (NICE) for decision-making, including both direct and indirect costs of low uptake and sub-optimal adherence. Other resources relevant to a wider societal perspective such as informal care and productivity loss (due to time off work) will be included in the secondary analyses.

Intervention costs will be estimated from information relating to staff time (RNs) in delivering the SUPA intervention, including time spent training and actually delivering the intervention, and other non-staff costs (e.g. manual development and printing). The frequency and duration of health and social care service use data will be combined with appropriate unit costs to generate total care costs per patient.

Costs will be compared between intervention and control groups. Bootstrapping methods will be used to produce confidence intervals around the cost differences to account for skewness often associated with the distribution of cost data. Costs will be combined with the primary trial outcome in the form of cost-effectiveness analyses. However, as the primary outcome is not a clinical outcome and is condition specific, quality adjusted life-years (QALYs) estimated from the EQ-5D will be used to estimate cost-effectiveness. The use of QALYs and the EQ-5D in HIV is supported by previous work. If the intervention results in reduced costs and better outcomes then it will be defined as being ‘dominant.’ However, supposing the costs are higher and outcomes are better, then incremental cost-effectiveness ratios will be calculated to show the extra costs incurred to gain an extra level of outcome. Uncertainty around cost-effectiveness estimates will be explored using cost-effectiveness planes. The results will be further evaluated using cost-effectiveness acceptability curves.

#### Determining the cost effectiveness of the long-term impact of the interventions

Although we will measure costs, QALYs and the cost per QALY over the study period, this information will be limited because it would be expected that QALY gains would largely occur later in time. To address this issue a Markov model will be constructed to examine how patients might move from one health state to another over a longer period of time. Health states will be defined according to CD4 counts or viral load. The probabilities of moving from one health state to another will be based on a review of the literature and from expert opinion. Assuming some degree of patient variation, the data collected in the trial will give information on EQ-5D utility scores and costs that are associated with different CD4 ranges and where necessary this information will be supplemented by data from the literature. As well as allowing us to assess the long-term cost-effectiveness of the interventions, this approach will enable an estimation of the long-term cost associated with different levels of ART uptake and adherence. This will be of importance from a public health perspective in that it will illustrate the economic savings that might be made through interventions. Increased uptake and adherence may have further benefits if infections in others are reduced and we will explore the possibility of incorporating such externalities into the model. The time horizon used will depend on data availability, but is it expected that we will measure costs and outcomes over a 5–10, and 15-year period. The results over the longer periods will by definition be more speculative.

### Internal feasibility review

Baseline data from the first 40 randomised patients (and 1-month data for those randomised to the intervention arm) will be reviewed with the Programme Management Group (PMG) and Programme Steering Committee (PSC) at respective group meetings. The groups will assess:

1. Feasibility of recruitment and retention, identifying barriers to recruitment/retention and problems in delivery.
2. Acceptability of study measures (Control and Intervention arms).
3. Acceptability of the intervention by patients (Intervention arm only).
4. Capacity of trial and local site personnel, including capacity of clinics to accommodate research staff, determining whether trial centres are fulfilling their commitments (i.e. helping with the identification and introduction of patients), and determining where further staff is needed to recruit patients and/or to deliver the intervention.

The findings of the feasibility review, along with participant feedback, will be used to make any necessary modifications to the conduct of the trial.

### Adverse events

Adverse events (AE) include any clinical change, disease or disorder experienced by the participant during their participation in the trial, whether or not considered related to participation in the trial. A Serious Adverse Event (SAE) will be defined according to usual clinical trial definitions. If the RA/RN is uncertain about whether the AE is an SAE, they will contact the centre Principal Investigator for their opinion. All SAEs must be reported by the RA/RN to the patient’s doctor (or doctor to the RA/RN), the centre Principal Investigator and the Trial Manager immediately. SAEs that are related to administration of any of the research procedures will be reported to the Sponsor and also sent to REC by the CI (or delegated individual) within 15 days of the CI becoming aware of the event.

After an SAE, a decision will be made as to whether the participant should be withdrawn from the trial, or need an alteration in their standard care. Arrangements will be made by the RN for further assessment and management as necessary. One month after an SAE, the RA/RN will provide both the site Principal Investigator and Trial Manager with a follow-up report. If the SAE is not resolved, further monthly reports will be sent via the Trial Manager to the Independent Data Monitoring Committee (IDMC). The RA/RN will forward these reports to the Research Ethics Committee, Sponsor, and local Research and Development (R&D) office.

### Trial discontinuation

If a member of the clinical care team or the RN feels that the intervention is detrimental (for example, causing distress), the patient can be referred out of the intervention. Referral to a more specialised professional should be carried out by the patient’s Clinician. Participants may discontinue from participation at any time, at the discretion of the Investigator. Specific reasons for discontinuing a participant from either study are:

1. Withdrawal of informed consent.
2. Development of exclusion criteria or other safety reasons during the study.
3. Incorrect enrolment or randomisation of the participant.

### Participation in other studies

Participation in other studies may be permitted (for example, qualitative or questionnaire-based studies) with the prior consent of the PMG. Patients who are participating in a study which encourages adherence (i.e. clinical trial of an investigational medical product) will be excluded.

### Strategies to promote recruitment

The following steps will be taken in order to achieve adequate participant enrolment

1. An RA will attend multidisciplinary clinical meetings at each site in order to identify study eligible patients.
2. Where possible an RA will be present at HIV clinics when ART-naïve patients have pre-booked appointments with a consultant and spoke with the consultant prior to the appointment in order to remind them of the patient’s eligibility for the trial. The RA will also attend emergency clinics in order to recruit patients attending clinics who are not on ART.
3. Written information has been developed to inform patients about different types of research (e.g. observational studies and randomised controlled trials). This is intended to help patients to make an informed decision about whether to take part in each part of the study.

### Strategies to promote data completeness

We will take the following steps in order to promote participant retention and complete follow-up:

1. Attempt to make the research visit convenient for participants by booking research visits to correspond with their regular care visits.
2. Participants will be contacted by their preferred mode of contact ahead of their research appointments to remind them and confirm attendance.
3. Participants will be offered the option of completing follow-up questionnaires by telephone.
4. Contact details will be checked at each appointment.
5. We will keep in touch with participants by sending a birthday card or text (provided the participant has consented to receive communication from us).

If the participant is withdrawn at the request of the Researcher or Clinician, final follow-up data should be collected as soon as possible (if appropriate). If the participant wishes to fully withdraw from Phase 1 or 2, the RA will contact the participant to ascertain the reason for withdrawal (although the patient does not have to give any reason), and ask whether they consent for previously collected data to be kept and analysed. The RA/RN will ensure that every effort is made to obtain any final follow up data (including MEMS caps). If the patient chooses to withdraw from attending the intervention only (not the trial), the RA should attempt to request permission to complete the further research follow-up visits (including keeping the MEMS caps) at the scheduled time points.

### Trial organisation and management

This study is being organised by the UCL School of Pharmacy, and sponsored by Brighton and Sussex University Hospitals NHS Trust. It is funded and has been peer-reviewed by the National Institute for Health Research (NIHR). Neither the sponsor nor the funding body were or will be involved in the study design, data collection, data analysis, interpretation of data, writing of the manuscript or the decision to submit the manuscript for publication.

The study has been given a favourable ethical opinion for conduct by the East of England–Essex Research Ethics Committee (13/EE/0235). It is overseen by the SUPA Trial Management Group (TMG), Programme Management Group (PMG), Independent Data Monitoring Committee (IDMC) and Programme Steering Committee (PSC). The trial was retrospectively registered with the ISRCTN (35514212) on 21/02/2014. Further details on the charter for each group can be found in Table 2.

**Table 2:** Trial organisation and management

Group	Contact details	
Sponsor	Trial Sponsor: Brighton and Sussex University Hospitals NHS Trust; Sponsor’s Reference 13/117/HOR; Contact name Scott Harfield; Address: Research & Development Directorate, Royal Sussex County Hospital, Eastern Road, Brighton, BN2 5BE Telephone: 01273 696,955 ext. 3538 E-mail: sponsorship.approvals@bsuh.nhs.uk	
	Main role	Specific responsibilities
Trial Management Group (TMG) (Chief investigator, Programme Managers)	Responsible for the design and conduct of SUPA and day to day management of the trial	-study planning -organisation of committee meetings-Provide annual report to funder -provide accrual figures to each site-Budget administration and contractual issues with individual centres-Advice for lead site investigators-Ethics committee and R&D applications-Data verification-Randomisation-Organisation of data storage -preparation of protocol and revisions to trial documents, -managing publication of study reports -maintaining databases, randomising patients, -ensuring complete and correct data -preparing reports -dealing with research governance
Programme Management Group (PMG)	Assisting in managing the trial, including the clinical and practical aspects	-input into and comment on the protocol and all trial documents -develop strategies to address any issues with recruitment -provide clinical or other expert guidance on clinical and practical queries and interpretation of information recorded on CRFs -input into the meetings of the PSC and IDMC when appropriate (open sections only) -provide responses for and consider the implications of any recommendations made by the IDMC and accepted by the PSC
Programme Steering Committee (PSC)	To act as the oversight body for this trial on behalf of the Sponsor/Funder	-provide expert oversight of the programme -make decisions as to the future continuation (or otherwise) of the programme -receive letters of feedback from the IDMC and consider their recommendations -assess the impact and relevance of any accumulating external evidence -monitor recruitment and follow-up rates and review strategies from PMG to deal with problems -approve any amendments to the protocol, where appropriate -approve any proposals by the PMG concerning any change to the design of the programme, including additional substudies -approve / comment on the statistical analysis plan, publication policy, main programme manuscript -approve external or early internal requests for release of data
Independent Data Monitoring Committee (IDMC)	Safeguard the interests of participants, assess the safety and efficacy of the interventions during the trial, and monitor the overall conduct of the trial. It is independent from the Sponsor.	-receive and review information on the progress and accruing data of this programme and provide advice on the conduct of the programme to the Programme Steering Committee (PSC) -inform the Chair of the PSC if, in their view the results are likely to convince a broad range of clinicians, including those supporting the programme and the general clinical community, that, on balance, one trial arm is clearly indicated -perform interim review of the programme’s progress including updated figures on recruitment, data quality, adherence to protocol treatment and follow-up, and main outcomes and safety data -monitor: evidence for treatment differences in the main efficacy outcome measures, evidence for treatment harm, recruitment figures and losses to follow-up, compliance with the protocol, sample size assumptions, compliance with previous IDMC recommendations, data quality and completeness -assess the impact and relevance of external evidence -suggest additional data analyses if necessary -advise on protocol modifications proposed by investigators
Lead Site Investigators	Responsible for local site trial management	In each participating centre a lead investigator (HIV consultant) will be identified, to be responsible for identification of patients, recruitment, data collection and completion of CRFs, along with follow up of study patients and adherence to study protocol at their local site.

### Ethics approvals

The research will be conducted in accordance with Good Clinical Practice and all applicable regulatory requirements including the Research Governance Framework and the Medicines for Human Use (Clinical Trial) Regulations 2004, 2006 and any subsequent amendments. Any modifications to the protocol will be agreed upon by the TMG and PMG, and approved by the REC.

### Trial status

The trial opened to recruitment in February 2014 and recruitment closed in June 2017, with both Phase 1 and 2 patients in follow up. No data cleaning or analysis of the trial has been executed prior to submission of this manuscript.

### Auditing trial conduct

Trial data inputed into the online database will be reviewed throughout the trial for accuracy and completeness by the programme manager(s) and a reviewer who has not been responsible for data collection. Recruitment figures from each site will be reviewed monthly by the programme manager(s) and supplied as a report quarterly to the Programme Management Group and monthly to site investigatorssite investigators. The IDMC will review recruitment figures and data completeness annually.

### Ancillary and post-trial care

Local approvals and indemnity will be sought by each participating centre through their local R&D department. Details of local indemnity arrangements ca1n be obtained through each centre’s NHS R&D department.

### Stopping guidelines

The IDMC will review blinded interim analyses by the IDMC annually. If the committee feels that the arms are unbalanced and being in one group is detrimental, the IDMC will advise the PSC who will review the findings and decide whether or not the trial should be stopped.

### Dissemination policy

Study findings will be disseminated through publication in peer-reviewed scientific journals, national and international conferences, HIV community publications and the NIHR Journals library.

## Discussion

We have described the design of the Supporting Uptake and Adherence to ART (SUPA) trial, an observational cohort study with nested randomised controlled trial evaluating the effectiveness of a CBT-based intervention to increase uptake and adherence to ART by addressing perceptual and practical barriers.

The intervention is based on the Necessity Concerns Framework and aims to address both perceptual and practical barriers to adherence as recommended by NICE [20] and BHIVA guidelines [9]. The intervention has been developed according to published guidelines for the development of complex interventions [15] and builds on preparatory research with PLWH showing that uptake and adherence to ART is driven by patients’ perceptions of their personal necessity for ART and concerns about adverse effects [18, 19]. To our knowledge, this is the first study to provide support at the time of a treatment recommendation to PLWH who may be at risk of delaying uptake or developing issues with adherence. This study targets individuals who may be considered as being hard to reach, and who are not usually represented in randomised trials. The study design allows the examination of process variables and the mechanism by which the intervention exerts its effect. The cost effectiveness analysis will enable health-care service providers to make informed decisions about the value of the intervention.

While it was not possible to mask the allocation of participants to intervention or control groups, we have taken steps to reduce bias, including blinding of the statistical team to group allocation and ensuring that data is not collected or entered by those delivering the intervention. It possible that the primary outcome measure (MEMS) may impact on adherence as it serves as a reminder to participants that they are monitored. However, the impact of adherence monitoring alone is likely to be minimal [36]. Although participants are selected for risk of delay to initiate treatment and nonadherence using a screening tool (BMQ-ART), patients who decline to take part in the study may represent a different group who are more at risk of disengaging from care. However, these participants will be followed up in the observational cohort, therefore their virological outcomes can be compared with those of trial participants.

This is the first study to apply the perceptions and practicalities approach recommended by NICE [20] to the design of an intervention to increase uptake and adherence to ART. It will provide information on the efficacy of the intervention as well as the mechanism by which the intervention exerts its effect. The findings will enable patients, healthcare providers and policy makers to make informed decisions about the value of the intervention.

## Additional files

Additional file 1: Summary of data collection at each timepoint (Phase 1 – observational study). (DOCX 14 kb)

Additional file 2: Summary of data collection at each timepoint (Phase 2 – trial). (DOCX 41 kb)

## Abbreviations

AE: Adverse events
ART: Antiretroviral therapy
bIPQ: Brief Illness Perception Questionnaire
BMQ-ART: Beliefs about Medicines Questionnaire - Antiretroviral therapy
CBT: Cognitive Behavioural Therapy
CSRI: Client Service Receipt Inventory
EQ-VAS: Euroqol Visual Analogue Scale
Euroqol-5D: EQ-5D-5 L
KCTU: King’s Clinical Trials Unit
MARS: Medication Adherence Report Scale
MEMS: Medication Event Monitoring System
MI: Motivational Interviewing
MRC: Medical Research Council
NHS: National Health Service
NICE: National Institute for Clinical Excellence
PAPA: Perceptions and Practicalities Approach
PLWH: People living with HIV
PMG: Programme Management Group
PSC: Programme Steering Committee
QALYs: Quality Adjusted Life-Years
R&D: Research & Development
RA: Research Assistant
RCT: Randomised controlled trial
REC: Research Ethics Committee
RN: Research Nurse
SAE: Serious Adverse Event
SAQ: Symptoms Associated with HIV and ART Questionnaire
SUPA: Supporting Uptake and Adherence to ART
TMG: Trial Management Group
