# Supplementary material for: Protocol of a two arm randomised, multi-centre, 12-month controlled trial: evaluating the impact of a Cognitive Behavioural Therapy (CBT)-based intervention Supporting UPtake and Adherence to antiretrovirals (SUPA) in adults with HIV
Source: BMC Public Health. 2019 Jul 8;19:905. doi: 10.1186/s12889-019-6893-z (PMC6615195; doi:10.1186/s12889-019-6893-z)
Supplement: Supplementary file 1 — Summary of data collection at each timepoint (Phase 1 – observational study). (DOCX 14 kb) [file 12889_2019_6893_MOESM1_ESM.docx]

Table 1 Summary of data collection at each timepoint (Phase 1 – observational study)

|  | **Baseline (month 0)** | **Month 3** | **Month 6** | **Month 12** |
| --- | --- | --- | --- | --- |
| Inclusion/Exclusion criteria | X |  |  |  |
| Statement of informed consent | X |  |  |  |
| Age | X |  |  |  |
| Sex | X |  |  |  |
| Ethnicity | X |  |  |  |
| Country of birth | X |  |  |  |
| Years lived in the UK | X |  |  |  |
| Parents’ country of birth | X |  |  |  |
| Sexual orientation | X |  |  |  |
| Marital status | X |  |  |  |
| Children | X |  |  |  |
| Children living at home | X |  |  |  |
| Living with people other than family? | X |  |  |  |
| Highest level of education | X |  |  |  |
| Current employment status | X |  |  |  |
| Household income | X |  |  |  |
| Date of HIV diagnosis | X |  |  |  |
| Country of diagnosis | X |  |  |  |
| Likely mode of transmission | X |  |  |  |
| Medical history (and changes to) | X | X | X | X |
| Concomitant diagnoses | X |  |  |  |
| Has the patient been prescribed ARVs? | X | X | X | X |
| Has the patient accepted their ARV treatment offer? | X | X | X | X |
| Date of first prescription for ARVs | X | X | X | X |
| Is the patient starting ARVs whilst being pregnant? | X | X | X | X |
| (If pregnant) how long for? (In weeks) | X | X | X | X |
| Which antiretroviral(s) has/have been prescribed? | X | X | X | X |
| How many doses per day? | X | X | X | X |
| Time of doses e.g. 13:00 (24-hour clock) | X | X | X | X |
| Purpose of concomitant medication | X | X | X | X |
| Frequency of concomitant medication e.g. once daily | X | X | X | X |
| Start date of concomitant medication | X | X | X | X |
| Is the participant registered with a GP? | X |  |  |  |
| GP aware of participant’s HIV status? | X |  |  |  |
| Disclosed HIV diagnosis to friend/relative (specify) | X | X | X | X |
| Know other people who are HIV positive? (e.g. friend) | X | X | X | X |
| Units of alcohol consumed per week? | X | X | X | X |
| Ever been a smoker? | X |  |  |  |
| How often does the participant use illicit drugs? | X | X | X | X |
| CD4 count | X | X | X | X |
| Viral load | X | X | X | X |
| Beliefs about Medicines Questionnaire (BMQ) | X | X | X | X |
