# Supplementary material for: Protocol of a two arm randomised, multi-centre, 12-month controlled trial: evaluating the impact of a Cognitive Behavioural Therapy (CBT)-based intervention Supporting UPtake and Adherence to antiretrovirals (SUPA) in adults with HIV
Source: BMC Public Health. 2019 Jul 8;19:905. doi: 10.1186/s12889-019-6893-z (PMC6615195; doi:10.1186/s12889-019-6893-z)
Supplement: Supplementary file 2 — Summary of data collection at each timepoint (Phase 2 – trial). (DOCX 41 kb) [file 12889_2019_6893_MOESM2_ESM.docx]

Table 2 Summary of data collection at each timepoint(Phase 2 – trial)

|  | **Baseline (month 0)** | **Month 1 (CBT only)** | **Month 3** | **Month 6** | **Month 12** |
| --- | --- | --- | --- | --- | --- |
| Inclusion/Exclusion criteria | X |  |  |  |  |
| Statement of informed consent | X |  |  |  |  |
| **Demographic data** | | | | | |
| Age | X |  |  |  |  |
| Sex | X |  |  |  |  |
| Ethnicity | X |  |  |  |  |
| Country of birth | X |  |  |  |  |
| Years lived in the UK | X |  |  |  |  |
| Parents’ country of birth | X |  |  |  |  |
| Sexual orientation | X |  |  |  |  |
| Marital status | X |  |  |  |  |
| Children | X |  |  |  |  |
| Children living at home | X |  |  |  |  |
| Living with people other than family? | X |  |  |  |  |
| Highest level of education | X |  |  |  |  |
| Current employment status | X |  |  |  |  |
| Household income | X |  |  |  |  |
| **Diagnosis** | | | | | |
| Date of HIV diagnosis | X |  |  |  |  |
| Country of diagnosis | X |  |  |  |  |
| Likely mode of transmission | X |  |  |  |  |
| Medical history (and changes to) | X | X | X | X | X |
| Concomitant diagnoses | X |  |  |  |  |
| **Medications** | | | | | |
| Has the patient been prescribed ARVs? | X | X | X | X | X |
| Has the patient accepted their ARV treatment offer? | X | X | X | X | X |
| Date of first prescription for ARVs | X | X | X | X | X |
| Is the patient starting ARVs whilst being pregnant? | X | X | X | X | X |
| (If pregnant) how long for? (In weeks) | X | X | X | X | X |
| Which antiretroviral(s) has/have been prescribed? | X | X | X | X | X |
| How many doses per day? | X | X | X | X | X |
| Time of doses e.g. 13:00 (24-hour clock) | X | X | X | X | X |
| **Concomitant Medications** | | | | | |
| Purpose of concomitant medication | X |  | X | X | X |
| Frequency of concomitant medication e.g. once daily | X |  | X | X | X |
| Start date of concomitant medication | X |  | X | X | X |
| **General Practice** | | | | | |
| Is the participant registered with a GP? | X |  |  |  |  |
| GP aware of participant’s HIV status? | X |  |  |  |  |
| **Social support** | | | | | |
| Disclosed HIV diagnosis to friend/relative (specify) | X | X | X | X | X |
| Know other people who are HIV positive? (e.g. friend) | X |  | X | X | X |
| **Alcohol and drug use** | | | | | |
| Units of alcohol consumed per week? | X |  | X | X | X |
| Ever been a smoker? | X |  |  |  |  |
| How often does the participant use illicit drugs? | X |  | X | X | X |
| **Laboratory data** | | | | | |
| CD4 count | X | X | X | X | X |
| Viral load | X | X | X | X | X |
| **Patient questionnaires** | | | | | |
| Beliefs about Medicines Questionnaire (BMQ) | X | X | X | X | X |
| ART intrusiveness scale (HIS) | X | X | X | X | X |
| Brief Illness Perceptions questionnaire (bIPQ) | X | X | X | X | X |
| Medication Adherence Report Scale (MARS) |  |  | X | X | X |
| Hospital Anxiety and Depression Scale (HADS) | X | X | X | X | X |
| Treatment readiness | X | X |  |  |  |
| Treatment knowledge | X | X | X | X | X |
| EQ-5D | X | X | X | X | X |
| Service use questionnaire | X |  | X | X | X |
| Symptoms Attribution Questionnaire (SAQ) | X | X | X | X | X |
| **Adherence (if started treatment)** | | | | | |
| Medication Adherence Report Scale (score) |  | X | X | X | X |
| MEMS data (how many times opened?) |  | X | X | X | X |
| What proportion of tablets was taken? (%) |  | X | X | X | X |
| What proportion of tablets was taken on time? (%) |  | X | X | X | X |
| **Intervention** | | | | | |
| Did they read the materials in their own time? |  | X | X | X | X |
| How useful did they find the materials? |  | X | X | X | X |
| How much did the materials made sense? |  | X | X | X | X |
| How easy did they were materials to read? |  | X | X | X | X |
| How enjoyable did they find were the materials? |  | X | X | X | X |
| How satisfied were they with the treatment support programme overall? |  | X | X | X | X |
| **Attendance of intervention services** | | | | | |
| No. contacts to reschedule intervention appointment |  | X | X | X | X |
| **ARV switches** | | | | | |
| Did participant request to switch ARVs? |  | X | X | X | X |
| Did physician switch ARVs? |  | X | X | X | X |
